# The Role of Rigidity in Adaptive and Maladaptive Families Assessed by FACES IV: The Points of View of Adolescents

**DOI:** 10.1007/s10826-016-0460-3

**Published:** 2016-06-03

**Authors:** Marina Everri, Tiziana Mancini, Laura Fruggeri

**Affiliations:** 1 Department of Social Psychology, London School of Economics, Houghton Street, WC2A 2AE London, UK; 2 LASS Department, University of Parma, Borgo Carissimi, 10, 43121 Parma, Italy

**Keywords:** Adolescence, Circumplex model, Rigidity, FACES IV, Latent class analysis

## Abstract

Previous studies using Olson’s Circumplex Model and FACES IV, the self-report assessing family functioning, did not clarify the role of rigidity, a dimension of this model. Rigidity emerged as ambiguous: it was considered either as a functional or as a dysfunctional dimension. Building upon the results of previous studies, we provided a contribution intended to disambiguate the role of rigidity considering adolescents’ perceptions and using a non-a priori classification analysis. 320 Italian adolescents (13–21 years) participated in this study and responded to a questionnaire containing scales of the study variables. A latent class analysis was performed to identify the association of rigidity with the other dimensions of Olson’s model and with indicators of adaptive family functioning in adolescence: parental monitoring and family satisfaction. We found six clusters corresponding to family typologies and having different levels of functioning. Rigidity emerged as adaptive in the typologies named rigidly balanced and flexibly oscillating; it was associated with positive dimensions of family functioning, i.e. flexibility, cohesion, parental monitoring, and high levels of family satisfaction. Differently, when rigidity was associated with disengagement, low cohesion and flexibility, and lack of parental supervision, emerged as maladaptive. This was the case of two typologies: the rigidly disengaged and the chaotically disengaged. Adolescents of these families reported the lowest levels of satisfaction. In the two last typologies, the flexibly chaotic and the cohesively disorganized, rigidity indicated a mid-range functionality as these families were characterized by emotional connectedness but lack of containment. Clinical implications are discussed.

## Introduction

The FACES IV (Family Adaptability and Cohesion Evaluation Scale) is the latest version of the family self-report used to assess the six dimensions of the Circumplex Model of Marital and Family Systems: cohesion, flexibility, disengagement, enmeshment, rigidity and chaos (Olson 2011; Olson and Gorall 2006; Olson, Russell and Sprenkle 1989; Olson et al. 1979). *Cohesion* and *flexibility* refers respectively to the family emotional bond, and to the family power, leadership, and rules; they are defined balanced dimensions as they assess a positive and well-functioning family environment. Differently, *disengagement* and *enmeshment* refer to either absent or excessive emotional bond, while *rigidity* and *chaos* refer to either strict or lax family power, leadership, and rules; they are defined unbalanced dimensions as they refer to a negative and maladaptive family environment. According to how these dimensions combine, different typologies of family functioning can be identified.

Previous studies in this field found a contrasting result for what specifically concerns the role of rigidity in defining the quality of the family functioning (e.g. Baiocco et al. 2013; Everri et al. 2015; Franklin et al. 2001). In fact, in Olson’s model rigidity indicates resistance to change, severe rules, rigid and highly differentiated family hierarchy, strong leadership and low adaptability, and it is negatively associated with the balanced dimensions of cohesion and flexibility. In the other studies, instead, rigidity was found to be positively associated with both the balanced dimensions of Olson’s model (cohesion and flexibility) and the parental supervision on children’s life, friends, and whereabouts, which is also an indicator of adaptive family functioning (Parental monitoring; Kerr et al. 2012; Stattin and Kerr 2000). This association emerges as contradictory to some extent given that adolescents seem to perceive their families as strict and severe, but also as flexible and cohesive, and monitoring their activities and private lives as well. Everri et al. (2015) advanced an explanation related to the socio-cultural background: being the participants of their study Italian adolescents, they “might have interpreted rigidity as a protective emotional bond related to more general parental engagement, e.g. awareness of their children’s activities, friends and interests” (p. 3064).

Previous studies validating FACES IV in different countries did not directly provided a similar interpretation of rigidity, however the few of them that considered samples of adolescents have outlined critical aspects related to rigidity assessment (for a review see: http://www.facesiv.com/home.html). For instance, Baiocco et al. (2013), who also studied Italian adolescents, found positive correlations between items of the rigidity subscale and items of the flexibility and of the cohesion subscales. Franklin and colleagues (Franklin et al. 2001) findings, obtained from a sample of American adolescents, indicated that the rigidity subscale did not have an adequate reliability and discriminant validity.

The main point of controversy highlighted by these works revolves around the association of the dimension of rigidity with indicators of either adaptive or maladaptive family functioning especially when adolescents’ perceptions are considered. A prospective way to illuminate the origin of rigidity ambiguity is the individuation of a more consistent empirical approach to data. Cluster analyses (Everitt et al. 2011), in particular, can serve the function. This procedure allows one to identify the empirical distribution of a set of variables in specific groups or typologies; consistently, it permits to explore how rigidity is distributed in the clusters derived from the combination of the adaptive (balanced: cohesion and flexibility) and maladaptive (unbalanced: disengagement, enmeshment and chaos) dimensions of FACES IV. The individuation of clusters, or family typologies in Olson’s model, is not new (Olson and Gorall 2006); however it has never been carried out before considering samples of adolescents.

In fact, in their original study, Olson and Gorall (2006) performed a K- means cluster analysis considering adults, and they identified six different family typologies that they named: *balanced, rigidly cohesive, midrange, flexibly unbalanced, chaotically disengaged,* and *unbalanced*. Families having high scores on cohesion and flexibility subscales and low scores on disengagement, enmeshment, rigidity and chaos subscales were defined as balanced. These families were considered adaptive, as they were able to handle daily living tasks and relational strains of changes in family over time. The midrange typology characterized families having a moderate level of functioning, given neither the high levels of strength and protective factors tapped by the balanced subscales, nor the high levels of difficulties or risk factors tapped by the unbalanced subscales. The flexibly unbalanced typology was considered the hardest to define clearly in terms of either adaptive or maladaptive functioning; these families presented high scores on all of the subscales other than cohesion, where moderate to low scores were observed. Olson and Gorall (2006) however noted that the high scores on the flexibility subscale may allow these families to alter problematic levels when necessary. A clearer interpretation was provided for both the chaotically disengaged and the unbalanced typologies; they characterized families with maladaptive functioning. Specifically, the chaotically disengaged were considered high problem families given the lack of emotional closeness, indicated by the low scores on cohesion subscales and the high scores on the disengagement subscales, and the high degree of problematic change indicated by the high scores on chaos subscale and low scores on flexibility subscale. Lastly, the unbalanced families were considered the most problematic in terms of their overall functioning as they presented: low scores on both cohesion and flexibility subscales and high scores on disengagement, enmeshment, rigidity and chaos subscales. These were considered clinical families.

More recent studies have replicated Olson and Gorall’s typologies using similar cluster analyses methods. For instance, Loriedo et al. (2013) considered a sample of Italian adults (mean age = 43.31) and identified five typologies, one of which, the rigidly balanced, did not overlap with those identified by Olson and Gorall as moderate levels of rigidity were positively related with both cohesion and flexibility. The authors considered this typology as moderately adaptive. Mirnics et al.’s study (2010), based on a sample of Hungarian adults (mean age = 43.50), found five typologies overlapping the Italian and the American typologies; the sixth, not named by the authors, was instead identified as peculiar of Hungarian culture, i.e. families with high tolerance for individual freedom and less enmeshment. In Spain, Rivero et al. (2010) found only four family typologies: balanced, chaotically disengaged, rigidly cohesive, and unbalanced, in a sample of young adults (mean age = 20.5). Thus, they did not find the midrange and the flexibly unbalanced typologies originally identified by Olson.

The results emerging from these studies highlight that the different number as well as the partially different content of the family typologies need to be analyzed more closely. We assume that the different number of the clusters found in each study is probably due to the traditional clustering applications used (k-means cluster and two steps cluster), substantially based on an arbitrary choice of cluster criterion (Magidson and Vermunt 2002). Ad hoc approach for classification and not statistical assistance in determining the number of clusters are in fact the main disadvantages of the clustering applications used in these studies. These disadvantages could be resolved by performing latent class methods (LCA) in respect of which renewed interest seems to emerge also in the psychological literature. LCA methods have some advantages over traditional cluster analysis applications and they have been found to outperform the traditional LC cluster models in several applications (Eshghi et al. 2011; Magidson and Vermunt 2001; 2002). LCA methods allow both a classification based on the posterior probability of belonging to each class and a classification assessment in terms of its quality. In LCA applications the number of classes are not defined a priori since various diagnostics such as the BIC and AIC statistic can be used in determining the optimal number of clusters. Also, the size of classes and misclassification rates can be controlled because of a model based on posterior membership probability estimated by maximum likelihood (ML) methods (Magidson and Vermunt 2002; Eshghi et al. 2011; Muthén and Muthén 2012).

As for the content of the typologies, rigidity emerged as an ambiguous dimension. In fact, high rigidity scores were present in two family typologies: in the rigidly cohesive that was found in the original Olson and Gorall study, and in the Hungarian and Spanish studies, and in the rigidly balanced that was found only in the Italian sample (Loriedo et al. 2013). As stated above, these typologies were not considered totally dysfunctional by these authors, however the high levels of rigidity in them could be problematic for families when they have to adapt to situational or developmental changes, such as in the case of adolescence.

In order to clarify the role of rigidity in the family typologies emerging from Olson’s model, we argue that two ways can be productively considered. First, we want to underline that the above results were obtained in samples of young adults/adults, and it was not reported whether they had partner and/or children and their age. This is not a marginal observation given that the positive correlations of rigidity with both the cohesion and flexibility dimensions of FACES IV have been found specifically in studies with adolescents (Baiocco et al. 2013; Everri et al. 2015). It is arguable therefore that a man in his forties having adolescent children has a different perception of rigidity as compared with a young single woman living with her family of origin. Thus, addressing our attention to more homogenous samples, for instance samples of adolescents, and considering the typologies emerging from adolescents’ perceptions, which have never been done before, can provide further insights on the rigidity dimension.

Second, the dimensions that constitute the family typologies can be connected to additional indicators of adaptive family functioning. The family studies literature has identified two important indicators, specifically for adaptive family functioning in adolescence, such as *parental monitoring* and *family satisfaction.* Parental monitoring concerns both the ability from parents’ part to supervise adolescent’s life, actively seek information from their adolescent children, negotiate family power, and the willingness from adolescents’ part to disclose about their private life to their parents (Stattin and Kerr 2000; Kerr et al. 2012). Moderate levels of parental monitoring during adolescence indicate well functioning families (Everri et al. in press; Henry et al. 2008; Mupinga et al. 2002).

Family satisfaction assesses how much individuals are satisfied of their family life. It was found that well-functioning families, such as the balanced typologies, presented also high levels of satisfaction; differently, the chaotically disengaged and the unbalanced typologies presented the lowest levels of satisfaction (Loriedo et al. 2013; Olson and Gorall 2006; Rivero et al. 2010). Also when considering samples of adolescents, a strong and positive correlation between the balanced dimensions of Olson’s model (cohesion and flexibility) and family satisfaction was observed (Baiocco et al. 2013).

Given the positive relations of parental monitoring and family satisfaction with dimensions of adaptive family functioning in samples of adolescents, it is arguable that according to adolescents’ perceptions moderate levels of parental monitoring and high levels of satisfaction will characterize adaptive family typologies. These relations could be usefully investigated to document whether rigidity dimension will characterize either these adaptive typologies or the maladaptive typologies in which perceived parental monitoring and family satisfaction should be low.

In sum, the present study had two specific aims: First, to determine the optimal number and the content of family typologies from the point of view of a sample of adolescents, thereby providing a better understanding of the role of rigidity in Olson’s Circumplex model, given also the controversy highlighted above. In order to do so, we used a non a priori classification analysis based on LCA. Second, to make an in-depth exploration of the content of the family typologies and of their level of functioning. In order to do so, we analyzed the family typologies considering the adolescents’ perception about both parental monitoring and family satisfaction.

## Method

### Participants

The study sample consisted of 320 adolescents: 144 boys and 175 girls, plus one case in which sex was not reported, aged between 13 and 21 years (M = 15.84, SD = 2.03). 183 adolescents attended the first year of high school (9th grade) and 137 attended the last year of high school (13th grade). Most adolescents were born in Italy (92.5 %), lived in two married parent households (251; 78.4 %), i.e. in traditional families, and had siblings (one sibling: 58.5 %; two siblings: 17.3 %; three or more siblings: 4.1 %). A non-negligible percentage of our adolescents (68, 21.3 %) lived in non-traditional families, i.e. families having either separated parents or stepparents.

### Procedure

Adolescents were recruited from two high schools in Northern Italy. Parents provided written consent for their children’s participation: none of the parents refused consent and all children decided to participate. Data collection was carried out in the classrooms over 1 h, in the presence of the teacher and the researcher who administered the questionnaire. Participation in the study was voluntary and anonymous, and participants were encouraged to answer individually and as truthfully as possible.

### Measures

#### Socio-Demographic Data

Some questions were used to collect information about: adolescents’ age, gender, household composition (number of family components) and family structure (e.g., cohabiting/married parents, separated parents, step-parents), and parents’ educational qualification and profession.

#### Family Functioning

The family adaptability and cohesion evaluation scale (FACES IV) was used to assess how adolescents perceived the functioning of their families. The Italian version based on the Olson’s last improvements added to FACES IV (Olson 2011) was validated by Baiocco et al. (2013) in a sample of adolescents and young adults. FACES IV contains 42 items that assess six dimensions on 7-items Likert-type scales (1-5 points); specifically two balanced subscales, cohesion and flexibility, assessing adaptive family functioning and four unbalanced subscales, enmeshment, disengagement, chaos and rigidity assessing maladaptive functioning. Items pertaining the dimensions of cohesion and flexibility concerned, respectively, the emotional bonding among family members (e.g. ‘‘In our family we like to spend our free time together’’) and the family leadership, rules, organization and negotiation (e.g. ‘‘In our family we have clear roles and rules’’). Sample items for the unbalanced subscales were: Enmeshment (e.g. “Family members feel pressured to spend most free time together”), disengagement (e.g. “Family members feel closer to people outside the family than to other family members”), chaos (e.g. “There is no leadership in this family”) and rigidity (“There are strict consequences for breaking the rules in our family”). In our study internal consistency was similar to the Italian validation (see Baiocco et al., 2013), we calculated the following alphas: cohesion (α = .78), flexibility (α = .70), enmeshment (α = .60), disengagement (α = .72), chaos (α = .56), and rigidity (α = .72).

#### Parental Monitoring

Adolescents’ perception of parental monitoring was assessed with the Parental Monitoring Questionnaire (Kerr et al. 2012; Stattin and Kerr 2000), validated in Italy by Miranda et al. (2012). This scale is composed of 25 items used to assess four different domains of parental monitoring (Everri et al. in press; Racz and McMahon 2011; Wang et al. 2013) on a five-point Likert scale where 1 indicates ‘not at all’, and 5 ‘always’. The four dimensions of parental monitoring and the internal consistency calculated in our study are: (a) *parental knowledge*, assessed with a nine-item subscale assessing perceptions of parents’ knowledge about one’s whereabouts, activities and peers (α = .70), (b) *youth disclosure*, assessed with a five-item subscale capturing adolescents’ tendency to provide unsolicited information (α = .77); (c) *parental control*, assessed with a six-item subscale containing items asking about whether the adolescent is required to inform parents about where he or she will be and with whom (α = .79); (d) *parental solicitation*, assessed with a five-item subscale relating to parental tendency actively to seek information about the adolescent (α = .72). The internal consistency of our study was acceptable if compared with the Cronbach alphas of Miranda and colleagues’ validation (see Miranda et al. 2012): parental knowledge (α = .86), youth disclosure (α = .76), parental control (α = .84), parental solicitation (α = .75). Specifically, it is measured considering four different domains: parental knowledge, parental control, parental solicitation, and youth disclosure.

#### Family Satisfaction

Family Satisfaction Scale (FSS) was developed by Olson (1995) in relation to the Circumplex model. We used the Italian adaptation by Baiocco et al. (2013). The scale assesses the degree of satisfaction with aspects related to family cohesion and flexibility, which was formulated in these items such as: “Your family’s ability to cope with stress”, “Your family’s ability to resolve conflict”, “Family members concern for each other”. The current version of the Family Satisfaction Scale contains 10 items on a Likert-type scale (α = .91) and is based on the original 14-item scale.

### Data Analyses

In order to disambiguate the role of rigidity in Olson’s Circumplex model and to determine the exact number of clusters emerged from adolescents’ perceptions, a LCA) was conducted using M-Plus 7.3.1 (Muthén and Muthén 2012). This method of analysis is generally used to explore how a set of unobserved subgroups of participants reliably differ in their points of view across a series of indicators (Hagenaars and McCutcheon 2002), which in our study were the Italian percentile scores on the six scales of FACES IV.

A multistage decision process combining fit statistics and substantive interpretability was chosen to decide the appropriate number of classes (Nylund et al. 2007). First, values of the Akaike information criteria (AIC) and Bayesian information criteria (BIC) were used to estimate the optimal number of classes (lower AIC and BIC values indicated better fitting models). Second, we used the entropy value (*E*) and the probability of a case belonging to each class as overall measures of the solution reliability and stability (values equal or major of .70 were considered adequate) (Murphy et al. 2007). Third, using the Bootstrap Lo-Mendell-Rubin test (BLRT; Nylund et al. 2007) with n = 500 iterations, we selected the solution that provided a significant improvement in the fit achieved in a solution with *k* − 1 classes. In order to choose the best class solution criteria, at least 1 % of the sample in the classes was considered as well as their conceptual distinction. Maximum likelihood estimation with robust standard errors (MLR) algorithm was used to create the classes on the basis of the level of variables inserted.

Family typologies derived from LCA were explored by gender, age, and family structure using Chi square test with Monte Carlo significance based on 10,000 sampled tables. Moreover, in order to make an in-depth exploration of both the family typologies content emerged from the LCA, and their level of functioning, a multivariate and a univariate analysis of variance were conducted using the statistical package for the social sciences (SPSS 21.0): Family typologies were considered as an independent factor and monitoring subscales and satisfaction scale as dependent variables.

## Results

Means, standard deviations and correlations between the study variables are reported in Table 1. The correlations showed that most variables were significantly correlated (*p* < .001). Cohesion and flexibility were highly and positively correlated (*r* = .61, *p* < .001) among themselves and negatively correlated with the unbalanced scales of disengagement and chaos. As previously found (see Everri et al. 2015), rigidity scale was appreciably associated with the balanced dimensions of cohesion (*r* = .20, *p* < .001) and flexibility (*r* = .37, *p* < .001), which indicates an adaptive family functioning. We also found that enmeshment was independent of most other variables although it was appreciably correlated with rigidity (*r* = .26, *p* < .001). Moreover it was not associated with either parental monitoring or family satisfaction.

**Table 1:** Descriptive statistics and bivariate correlations for all variables (N = 320)

	Alpha	M	SD	1.	2.	3.	4.	5.	6.	7.	8.	9.	10.	11.
1. Cohesion (Perc.)	.78	61.20	27.61	1.00										
2. Flexibility (Perc.)	.70	69.96	26.44	.613***	1.00									
3. Disengagement (Perc.)	.72	66.44	25.59	−.565***	−.450***	1.00								
4. Enmeshment (Perc.)	.60	36.26	23.50	−0.11	0.06	0.08	1.00							
5. Rigidity (Perc.)	.72	74.67	24.15	.202***	.371***	−.111*	.260***	1.00						
6. Chaos (Perc.)	.56	69.37	22.52	−.151**	−.174**	.183**	0.10	−.224***	1.00					
7. Parental Knowledge (1–5)	.70	3.63	0.58	.479***	.321***	−.334***	0.03	.271***	−.134*	1.00				
8. Youth Disclosure (1–5)	.77	3.34	0.80	.460***	.355***	−.382***	0.02	0.09	−0.02	.547***	1.00			
9. Parent Control (1−5)	.79	3.58	0.92	.211***	.186**	−.219***	0.11	.387***	−.181**	.437***	.153**	1.00		
10. Parent Solicitation (1−5)	.72	3.21	0.81	.482***	.393***	−.314***	−0.04	.194***	−0.10	.557***	.476**	.322***	1.00	
11. Family Satisfaction	.91	3.42	0.80	.661***	.601***	−.519***	−0.05	.237***	−.171**	.418***	.407**	.187**	.427***	1.00

Mean differences in the six components of FACES IV across the gender and age did not revealed significant multivariate effects: Wilks’ *λ*_Gender_ = 0.97, *F*(6312) = 1.75, *p* > .05, *η*_*p*_^2^ = .03; Wilks’ *λ*_Age_ = 0.96, *F*(6313) = 1.89, *p* > .05, *η*_*p*_^2^ = .04. There was instead a significant but weak multivariate effect among family structures on the six components of FACES IV: Wilks’ *λ* = 0.95, *F*(6312) = 2.89, *p* < .01, *η*_*p*_^2^ = .05. Univariate results evidenced significant differences on rigidity for all the three independent variables, *F*_*Gender*_ (1, 319) = 4.20, *p* < .05, *η*_*p*_^2^ = .01; *F*_*Age*_ (1, 320) = 4.33, *p* < .05, *η*_*p*_^2^ = .01; *F*_*Family structure*_ (1, 319) = 6.98, *p* < .01, *η*_*p*_^2^ = .02, with male, 9th grade, and living in traditional families, adolescents scored higher than female, 13th grade, and living in not-traditional families. Adolescent lived in traditional families also reported higher scores on cohesion, *F* (1, 319) = 8.46, *p* < .01, *η*_*p*_^2^ = .03, and flexibility, *F* (1, 319) = 11.41, *p* < .01, *η*_*p*_^2^ = .04, compared with peers lived in not-traditional families.

Parental monitoring dimensions are all significantly correlated. As for the relationships with family functioning scales, we observed the same trend for parental knowledge, parental control and parental solicitation: positive correlation with the balanced dimensions (cohesion and flexibility), including rigidity, and negative correlation with disengagement and chaos. Youth disclosure correlations presented the same trend, however no relation was observed with rigidity.

Mean differences in the four components of parental monitoring across gender, age and family structure revealed significant multivariate effects for gender, Wilks’ *λ* = 0.94, *F*(4314) = 5.41, *p* < .001, *η*_*p*_^2^ = .06, and for age, Wilks’ *λ* = 0.76, *F*(4315) = 25.49, *p* < .001, *η*_*p*_^2^ = .25, but not for family structure, Wilks’ *λ* = 0.97, *F*(4314) = 2.06, *p* > .05, *η*_*p*_^2^ = .03. Univariate results evidenced that female scored higher than male in all the four monitoring dimensions, *F*_*Parental Knowledge*_ (1, 319) = 6.40, *p* < .05, *η*_*p*_^2^ = .02; *F*_*Youth Disclosure*_ (1, 319) = 14.92, *p* < .001, *η*_*p*_^2^ = .05; *F*_*Parental Control*_ (1, 319) = 8.90, *p* < .01, *η*_*p*_^2^ = .03; *F*_*Parental Solicitation*_ (1, 319) = 6.92, *p* < .01, *η*_*p*_^2^ = .02; 9th grade scored higher than 13th grade participants on Parental Knowledge, *F* (1, 320) = 4.88, *p* < .05, *η*_*p*_^2^ = .02, and Parental Control, *F*(1, 320) = 69.20, *p* < .001, *η*_*p*_^2^ = .18, and, vice versa 13th grade participants scored higher than 9th grade in Youth Disclosure, *F*(1, 320) = 7.12, *p* < .01, *η*_*p*_^2^ = .02. Finally, adolescent lived in traditional families reported higher scores on Parental Knowledge, *F* (1, 319) = 7.68, *p* < .01, *η*_*p*_^2^ = .02, compared with peers lived in not-traditional families.

Lastly, family satisfaction was positively related to most of the study dimensions, negatively related with disengagement and chaos, and it was not related with enmeshment. Moreover, family satisfaction did not vary according to the adolescents’ gender, *F*(1, 317) = 0.31, *p* > .05, *η*_*p*_^2^ = .05, and age, *F*(1, 318) = 1.65, *p* > .05, *η*_*p*_^2^ = .01; instead adolescents living in traditional families reported higher scores on family satisfaction, *F* (1, 317) = 7.92, *p* < .01, *η*_*p*_^2^ = .02, compared with peers living in not-traditional families.

We examined a range of different cluster solutions of LCA with models ranging from 2 to 7 latent classes. The information criteria AIC and BIC favored the six-class solution: AIC criteria decreased (two-class = 17 488.43; three-class = 17 388.11; four-class = 17 313.84; five-class = 17 245.89; six-class = 17 205.88; seven-class = 17 191.87) and BIC criteria increased (two-class = 17 560.03; three-class = 17 486.20; four-class = 17 438.20; five-class = 17 396.62; six-class = 17 382.99; seven-class = 17 395.36), once the six latent classes had been specified. This suggested that modeling additional classes beyond six did not meaningfully improve the model. The six latent classes solution was reliable enough (*E* = .90). The classification accuracy rates indicated excellent classification likelihood and only a small average of misclassification biases and samples size for all the six classes were: c1 = .95; c2 = .97; c3 = .93; c4 = .90; c5 = .93; c6 = .90. Sample sizes for the classes were all > of 1 %: c1 = 27; c2 = 11; c3 = 79; c4 = 49; c5 = 122; c6 = 32. The Lo-Mendell-Rubin adjusted likelihood ratio test (aLRT) supported a six-class solution. The six-class solution fit the data significantly better than an alternative five-class solution (aLRT = 52.71. *p* < .05). However a seven-factor solution did not produce a significant improvement beyond the six-class model (aLRT = 27.33. *p* = .57). Bootstrapped parametric likelihood ration test confirmed this result (*p* < . 001).

**Fig. 1:**
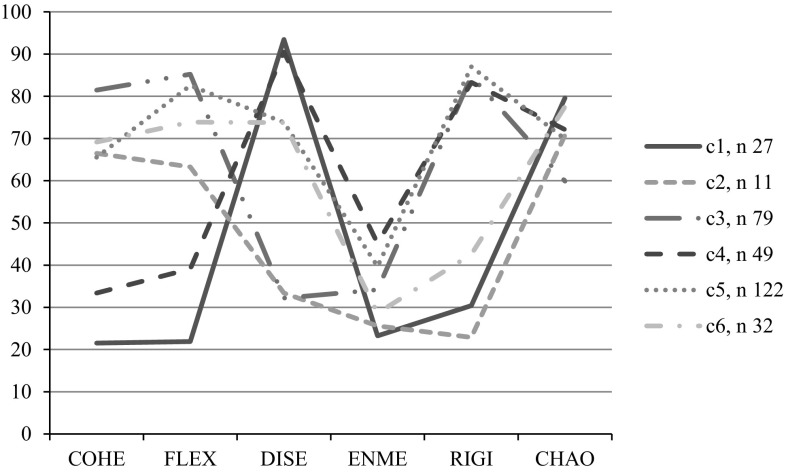
Estimated means in Cohesion (COHE), Flexibility (FLEX), Disengagement (DISE), Enmeshment (ENME), Rigidity (RIGI), and Chaos (CHAO) for a six-class solution estimated using LCA (Adaptation from Loriedo et al. 2013)

Estimated means for the level of cohesion, flexibility, disengagement, enmeshment, rigidity, and chaos for these six different typologies or classes of families according to the points of view of adolescents are presented in Fig. 1 below.

Two classes (c1 and c4) out of six showed low levels of cohesion and flexibility, and high levels of disengagement and chaos; nevertheless, they differed in enmeshment and rigidity levels: c4 class scored moderate in enmeshment and high in rigidity, while c1 scored low on both these dimensions. These classes were consistent with predictions derived from both Olson model and empirical literature, which define them as unbalanced family typologies characterizing maladaptive families, given the high levels of both disengagement and chaos. In particular, cluster 4 is characterized by high levels of rigidity so that it can be defined as *rigidly disengaged*, while cluster 1 is characterized by low levels of rigidity and high levels of chaos so that it can be named *chaotically disengaged*. According to our analysis, a relatively small proportion of adolescents (8.4 %) perceived their families as chaotically disengaged, while a larger proportion (15.3 %) perceived their families as rigidly disengaged.

The analysis also identified two clear, but not univalent, balanced classes (c2 and c3). They showed either high (c3) or medium–high (c2) levels of cohesion and flexibility, low levels of disengagement and enmeshment, and high levels of chaos. However, these classes differed in the rigidity dimension levels: c3 class scored high while c2 scored low. It was interesting to note that in contrast to the predictions derived from Olson’s model, the balanced family typologies, which characterized about a quarter (c3 = 24.7 %) of our participants, presented high levels of rigidity. Adolescents perceived their families as enough cohesive and flexible, thus balanced, but also highly rigid; thus cluster 3 can be named *rigidly balanced*. A relatively small proportions of adolescents (c2 = 3.4 %), instead, perceived their families as moderately highly cohesive and flexible, but marginally rigid. Slightly higher levels of chaos than the rigidly balanced characterize this family class, thus we labeled it: *cohesively disorganized.*

A mid-range profile seemed to characterize the last two classes (c5 and c6) that enclosed about the 50 % of participants. These classes showed medium–high levels of cohesion and high levels of flexibility, medium–high levels of disengagement, and medium–low levels of enmeshment. Nevertheless, as in the classes described above, they differed in terms of rigidity and chaos levels: c5 class scored high and c6 class scored medium–low on rigidity, and c5 class scored lower on chaos than c6 class. Given that these family typologies have high scores on the flexibility dimension, we labeled them respectively, *flexibly oscillating* (that constitute 38.1 % of the participants) and *flexibly chaotic* (that constitute 10.0 % of the participants). It was interesting to note the contradiction of high scores on both flexibility and rigidity in the flexibly oscillating typology, which is also characterized by the lowest level of chaos. In contrast, it was the high levels of chaos and the moderate levels of rigidity associated with high levels of flexibility that distinguished the flexibly chaotic class.

The analysis of the different family typologies considering socio-demographic variables (gender, age, and family structure), parental monitoring and family satisfaction are presented in Table 2 below.

**Table 2:** Number and percentage of adolescents in the six family typologies according to gender, age, family structure, and mean differences and standard deviations of the six typologies according to parental monitoring domains and family satisfaction

	1 Chaotically disengaged		2 Cohesively disorganized		3 Rigidly balanced		4 Rigidly disengaged		5 Flexibly oscillating		6 Flexibly chaotic		Total	
	*N*	*%*		*%*	*N*	*%*	*N*	*%*	*N*	*%*	*N*	*%*	*N*	*%*
Gender														
Male	8	5.6 %	4	2.8 %	32	22.2 %	23	16.0 %	64	44.4 %	13	9.0 %	144	100.0 %
Female	19	10.9 %	7	4.0 %	47	26.9 %	25	14.3 %	58	33.1 %	19	10.9 %	175	100.0 %
Age														
9th grade	12	6.6 %	7	3.8 %	46	25.1 %	25	13.7 %	79	43.2 %	14	7.7 %	183	100.0 %
13th grade	15	10.9 %	4	2.9 %	33	24.1 %	24	17.5 %	43	31.4 %	18	13.1 %	137	100.0 %
Family structure														
Non-traditional families	12	17.6 %	3	4.4 %	12	17.6 %	13	19.1 %	22	32.4 %	6	8.8 %	68	100.0 %
Traditional families	15	6.0 %	8	3.2 %	67	26.7 %	36	14.3 %	99	39.4 %	26	10.4 %	251	100.0 %

	*M*	*SD*	*M*	*SD*	*M*	*SD*	*M*	*SD*	*M*	*SD*	*M*	*SD*	*M*	*SD*
Parental monitoring														
Parental knowledge	3.20	.68	3.44	.89	3.95	.41	3.43	.54	3.66	.49	3.47	.66	3.63	.58
Youth disclosure	2.72	.88	3.35	.92	3.78	.67	2.92	.65	3.35	.76	3.39	.72	3.34	.80
Parental control	3.04	1.06	2.94	.90	3.97	.76	3.55	.89	3.66	.86	2.99	.93	3.58	.92
Parental solicitation	2.65	.89	2.94	1.01	3.60	.66	2.74	.76	3.30	.77	3.19	.66	3.21	.81
Family satisfaction	2.25	.77	3.65	.99	3.94	.58	2.91	.76	3.51	.57	3.48	.56	3.42	.80

As shown in Table 2, males were more likely than females to describe their family as flexibly oscillating (standardized adjusted residual = 2.1), but no other significant differences emerged (*χ*^2^ (5) = 6.70, *p*_*e*_ = .25). Younger adolescents (9th grade) were more likely to describe their family as flexible oscillating (standardized adjusted residual = 2.1) than did adolescents attending 13th grade. No other significant differences emerged (*χ*^2^ (5) = 7.99. *p*_*e*_ = .15). Participants living in non-traditional families were more likely than those living in traditional families to describe their family as chaotically disengaged (standardized adjusted residual = 3.1; *χ*^2^ (5) = 12.24. *p*_*e*_ = .03). No other significant differences emerged comparing the other five classes.

Table 2 also presents the mean differences and standard deviations in the four components of parental monitoring across the six family classes. It revealed a significant multivariate difference among family typologies: Wilks’ *λ* = 0.69, *F*(20, 1032) = 6.08, *p* < .001, *η*_*p*_^2^ = .09. Univariate results evidenced significant differences on all the four dimensions of parental monitoring: *F*_*Parental Knowledge*_ (5, 319) = 11.70, *p* < .001, *η*_*p*_^2^ = .16; *F*_*Youth Disclosure*_ (5, 319) = 12.61, *p* < .001, *η*_*p*_^2^ = .17; *F*_*Parental Control*_ (5, 319) = 9.62, *p* < .001, *η*_*p*_^2^ = .13; *F*_*Parental Solicitation*_ (5, 319) = 11.66, *p* < .001, *η*_*p*_^2^ = .16.

Post-hoc analysis (Tukey test) revealed that adolescents in the chaotically disengaged family typology scored lower on all the four dimensions of parental monitoring than did adolescents in the *rigidly balanced* (*p* < .001) and in the flexibly oscillating (*p* < .01) typologies. Adolescents in chaotically disengaged families also scored lower on youth disclosure monitoring dimension (*p* < .01) than did adolescents in the flexibly chaotic typologies.

Adolescents in the rigidly balanced family typologies scored higher than did adolescents in the rigidly disengaged typologies on: parental knowledge (*p* < .001), youth disclosure (*p* < .001) and parental solicitation (*p* < .001). They also scored: higher on parental knowledge (*p* < .01) and youth disclosure (*p* < .01) than did adolescents in the flexibly oscillating families; higher on parental knowledge (*p* < .001) and on parental control (*p* < .001) than did adolescents in the flexibly chaotic typologies; higher on parental knowledge (*p* < .05) and youth disclosure (*p* < .01) than did adolescents in the cohesively disorganized typologies. Adolescents in flexibly oscillating families scored higher on youth disclosure (*p* < .01) and parental solicitation (*p* < .001) than did adolescents in the rigidly disengaged typologies; they also scored higher on parental control (*p* < .01) than did adolescents in the flexibly chaotic family typologies.

Paired t-student analyses conducted within each class showed that parental knowledge was significantly (*p* < .05 or .01 or .001) higher than both youth disclosure and parental solicitation in the rigidly balanced, flexibly oscillating, chaotically disengaged and rigidly disengaged family typologies. In these family typologies, parental control was also significantly higher than parental solicitation. Only in the cohesively disorganized and in the flexibly chaotic family typologies parental knowledge was significantly higher than parental control. Parental control was significantly higher than youth disclosure in the flexibly oscillating and rigidly disengaged family typologies, while the reverse occurred in the flexibly chaotic families. Lastly, youth disclosure was significantly higher than parental solicitation in the rigidly balanced family typology.

Results of an analysis of variance revealed a significant difference among family types on family satisfaction: *F*(5314) = 35.49, *p* < .001, *η*_*p*_^2^ = .36. The post hoc analysis (Tukey test) indicated that adolescents in chaotically disengaged family type scored lower on family satisfaction (*p* < .001) than did adolescents in all the other family classes. Adolescents in the rigidly balanced family typologies scored higher than did those in the rigidly disengaged (*p* < .001), flexibly oscillating (*p* < .001), and flexibly chaotic (*p* < .01) family typologies. Nevertheless, the flexibly oscillating (*p* < .001), and flexibly chaotic (*p* < .01) scored higher than did the rigidly disengaged, which in scored higher than did the cohesively disorganized typologies (*p* < .01).

## Discussion

The present paper was intended to better understand family functioning in adolescence considering adolescents’ points of view, and adopting Olson’s Circumplex model as theoretical framework. In previous studies, rigidity, one dimension of this model, resulted ambiguous: In fact, they did not clarify whether rigidity had to be considered either as an adaptive dimension or as a maladaptive dimension. The results of the present study permitted to partially disambiguate the role of rigidity.

Using a non-a-priory cluster analysis, i.e. Latent Class Analyses (LCA), we found six different family typologies in which rigidity was associated with both the functional and the dysfunctional dimensions of Olson’s model, and with other variables of adaptive family functioning in adolescence, i.e. parental monitoring and family satisfaction. Previous studies (e.g. Loriedo et al. 2013; Mirnics et al. 2010; Olson and Gorall 2006) indicated that adaptive family typologies, which were defined as balanced, are characterized by high levels of positive balanced dimensions (cohesion and flexibility) and low levels of negative unbalanced dimensions (disengagement, enmeshment, chaos), also including rigidity in them. Instead, in our results rigidity did not emerge as negative *per sè:* Its positive or negative role depended on the positive or negative dimensions of family functioning it was associated with.

The six family typologies emerged from our analyses were: rigidly balanced, flexibly oscillating, flexibly chaotic, cohesively disorganized, rigidly disengaged, and chaotically disengaged. In order to make these typologies comparable with those identified in previous studies using FACES IV, we decided to use similar labels. We found that these family typologies did not substantially differ in terms of adolescents’ age, gender, and family structure; instead, they differed in the extent to which they illuminated family dynamics that could be either positive and functional or negative and dysfunctional for the developmental tasks that characterize adolescence.

The highest levels of rigidity were found in the rigidly balanced and in the flexibly oscillating typologies, which emerged as the most adaptive and as the most frequent in our sample. In fact, in both these typologies rigidity was associated with positive dimensions of family functioning, such as cohesion, flexibility, high levels of parental monitoring, and high levels of family satisfaction. The rigidly balanced typology was also found in a sample of Italian adults (Loriedo et al. 2013), but it was considered moderately adaptive. In our sample, adolescents perceiving their families as *rigidly balanced* also reported that their parents had a high knowledge and a high control of their private life (parental knowledge), and that they openly shared private information (youth disclosure) that parents stimulated (parental solicitation). As reported by different studies, parental knowledge is highly protective for adolescents’ adjustment (Crouter et al. 2005; Laird et al. 2007; Neumann et al. 2010). Notwithstanding the averages of parental monitoring measures in the rigidly balanced typology were higher than in the other typologies, we observed that it presented higher levels of parental control than of parental solicitation, and that parental solicitation was lower than youth disclosure. Therefore, in this typology we found high levels of control, which according to adolescents seemed to be valued as important, at least at the moment, given the high satisfaction of their families.

The *flexibly oscillating,* the most represented typology of our sample (38.1 %), showed high levels of rigidity, flexibility and disengagement together with low levels of cohesion. For this kind of association among the dimensions, we interpreted this typology as possibly typical of a period of intense family re-organization, i.e. adolescence. The flexibly oscillating was mainly observed in male and younger adolescents (9th grade), that is, the group of adolescents that are in the middle of the process that leads to their individuation. They seemed to perceive themselves as part of a system, their family, which oscillated between closeness (cohesion) and distance (disengagement), low leadership and rules (flexibility), but also protection and parental engagement (rigidity). This typology overlapped with other studies in which it was found that the oscillation between the different aspects of family functioning is the process that in adolescence allows families to incorporate incoming changes in their interactive family repertoires (Molinari et al. 2010; Everri et al. 2014). The same group of adolescents referred to perceive their parents as moderately high monitoring, and to have high levels of satisfaction with respect to their families.

Differently, the *rigidly disengaged* typology had high levels of rigidity that were associated with negative dimensions of family functioning. Specifically, this typology presented: low cohesion and flexibility, levels of youth disclosure and parental solicitation below the theoretical median of the scale, moderately high levels of parental control and parental knowledge, and low levels of adolescents’ satisfaction of their families. Adolescents belonging to this kind of families drew a scenario that indicated a critical developmental context in which they seemed to perceive themselves as separated from the other family members. Thus, adolescents perceived rigidity as a lack of parental engagement in their life. Given these characteristics, these families can be associated to those defined by the literature as disengaged families (Cox and Paley 1997; Minuchin 1974; Sturge-Apple et al. 2010), which were related to the children’s development of externalizing problems.

Rigidity emerged as low in the *chaotically disengaged* family typology, in which it was associated with low levels of both cohesion and flexibility, and high levels of both disengagement and chaos. Adolescents seemed to interpret low levels of rigidity as distance, and lack of rules and leadership; these aspects were also associated with scarce attention from parents’ part to adolescents’ life, and absence of adolescents’ disclosure. Moreover, adolescents of this family typology presented the lowest levels of family satisfaction with respect to the other groups of adolescents. Olson and other scholars (Loriedo et al. 2013; Mirnics et al. 2010; Rivero et al. 2010; Olson and Gorall 2006) also found this typology in samples of adults: According to them, chaotically disengaged families are highly problematic. In this line, these families can be considered as non-adaptive contexts also for adolescents, especially for the lack of emotional closeness and of clear boundaries among family members, together with the lack of interest of parents in their children’s life.

Moderate-low and low levels of rigidity were also observed in the *flexibly chaotic* and in the *cohesivelydisorganized* typologies. In these cases, rigidity was associated with high levels of chaos, but also with high levels of flexibility and cohesion, and moderately high levels of satisfaction. Interestingly, in these typologies we found higher levels of parental knowledge and youth disclosure than of parental control and solicitation. These two groups of adolescents seemed therefore to indicate that they belonged to families in which the communication with their parents is open, so that they felt comfortable in telling them private issues of their life. Adolescents also seemed to report that the emotional bond and the attention on behalf of parents were connected to low levels of rules and leaderships and to high levels of disorganization; so they perceived good emotional closeness, which was however associated with lack of containment. Despite this aspect, adolescents in these families were moderately satisfied; this was probably due to the perception of being emotionally close to their parents and to the possibility of openly communicate with them. Given these characteristics, these family typologies can be considered typical of contemporary families, in which the continuum norms-affection seems to lean toward the affection part (Williams 2004).

Besides theoretical and empirical advances in the field of Olson’s Circumplex Model and FACES IV research with adolescent samples, these findings can provide hints for clinical practitioners working with adolescents and families. It is likely that families characterized by high levels of disengagement, such as the chaotically disengaged and the rigidly disengaged family typologies, encounter practitioners especially during adolescence, when families need to be flexible enough to favor adolescents’ individuation, but without loosing emotional connectedness. Adolescents in these families can also be more likely to develop problematic behaviors. Practitioners can productively benefit of adolescents’ points of view for better identifying families’ critical aspects of their functioning: As showed by our results, adolescents seem to demand more engagement, emotional connectedness and containment from their parents. Families can therefore be empowered giving voice to their adolescent children, and use what they feel as a resource for their clinical treatments.

Some limitations can be found to this study. First, it is important to note that our results are based on a cross-sectional analysis of the study variables; therefore, we cannot confirm whether the family typologies are characteristic either of the adolescence phase, thus they are subject to change, or of the general family functioning, thus whether they are relatively stable independently from the developmental stage considered. A longitudinal analysis could clarify this aspect. Second, the observed family typologies were found considering only adolescents’ perceptions. Collecting data on parents’ perceptions would provide a more comprehensive understanding or the family typologies that we have identified. Lastly, in our study we only included variables related to how families function as a whole; instead, assessing individual aspects related to adolescents’ adjustment would be more informative of the relationship between the family typologies and their impact on specific aspects of adolescents’ development.
